# Supplementary material for: Significant Serpents: Predictive Modelling of Bioclimatic Venom Variation in Russell’s Viper
Source: PLoS Negl Trop Dis. 2025 Apr 10;19(4):e0012949. doi: 10.1371/journal.pntd.0012949 (PMC11984747; doi:10.1371/journal.pntd.0012949)
Supplement: S1 File — Fig A. Statistics of MLR models with AIC. Table A. Sampling location and bioclimatic variables. Table B. Statistics of GWR and MLR (Global) Models. Table C. Statistics of SLR models. (PDF) [file pntd.0012949.s001.pdf]

Figure A: Statistics of MLR models with AIC. Various MLR models were built to explain the variation in the *D. russelii* venom PLA_2, protease and LAAO activities using the prevailing bioclimatic factors at the sampling locations. A combination of independent variables (IV), as depicted by yellow squares, was found to significantly affect the venom activities (A[enzyme]). The purple squares indicate variables that did not significantly contribute to the model. Logarithmic [ln(activity)], square-root [(activity)½], and inverse [1/(activity)] transformations of the dependent variables (DV) were also performed. The blue squares highlight the models that were downselected. Various model tests were also performed to assess normality (N), homoscedasticity (H), Linearity (L) and Multicollinearity (M) of the models. The green and red squares highlight if a particular MLR model passed or failed the corresponding model tests, respectively. The details of the bioclimatic variables included in the models are provided in Table 1. AMT: Annual mean temperature, TAR: Temperature annual range, AMDTR: Annual mean diurnal temperature range, I: Isothermality, TS: Temperature seasonality, APN: Annual precipitation and PS: Precipitation seasonality. AIC values of the models have also been included to provide robust statistical support along with R^2 and p-value.

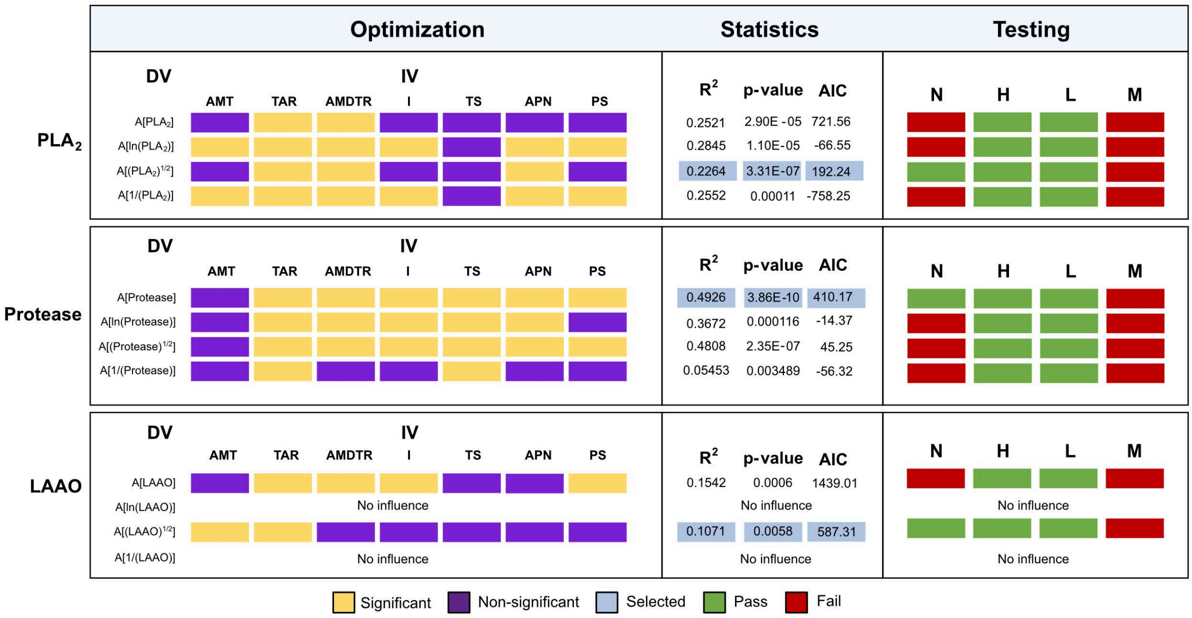

Table A: Sampling location and bioclimatic variables. The table presents the list of samples collected from various location across the country. The information on pooling of samples, protein concentration of venoms as well as the enzymatic activities are provided. The bioclimatic variables retrieved using the latitudes and logitudes of the corresponding sampling locations are also included.

Sl No	Sample ID	State	Location	Individuals	Protein Concentration	Average LAAO activity (nmol/mg/min)	AveragePLA2 Activity (nmol/mg/min)	Average Relative protease activity (%)	Latitude	Longitude	Annual Mean Temperature (AMT)	Mean Diurnal Range (AMDTRR)	Temperature Annual Range (TAR)	Temperature Seasonality (TS)	Isothermality (I)	Annual Precipitation (APNN)	Precipitation Seasonality (PS)
1	Pb01	Punjab	Nawanshar	2	3.831	1729.293	106.926	1.42	31.13	76.12	23.42083	12.55833	33.1	716.6001	37.94059	834	122.16037
2	Rj01	Rajasthan	Udaipur	1	3.441	918.967	15.034	50.66	24.59	73.7	24.9125	12.04167	29.3	506.25238	41.09784	639	141.93684
3	Rj02	Rajasthan	Udaipur	1	3.129	1392.593	14.696	49.29	24.59	73.7	24.9125	12.04167	29.3	506.25238	41.09784	639	141.93684
4	Mp01	Madhya Pradesh	Jabalpur	1	3.311	4730.415	105.687	45.80	23.18	79.98	25.10417	12.80833	31.8	535.23767	40.27778	1265	146.35542
5	Mp02	Madhya Pradesh	Jabalpur	3	3.402	4346.577	162.782	47.11	23.18	79.98	25.10417	12.80833	31.8	535.23767	40.27778	1265	146.35542
6	Mp03	Madhya Pradesh	Jabalpur	1	3.121	4880.808	99.944	51.88	23.18	79.98	25.10417	12.80833	31.8	535.23767	40.27778	1265	146.35542
7	Mp04	Madhya Pradesh	Bhopal	1	3.558	7787.654	101.408	47.82	23.24	77.4	25.46667	12.36667	29.1	472.62961	42.49714	1171	148.83801
8	Mp05	Madhya Pradesh	Bhopal	1	3.253	5592.368	109.403	49.73	23.24	77.4	25.46667	12.36667	29.1	472.62961	42.49714	1171	148.83801
9	Mh01	Maharashtra	Mahad	5	4.028	6146.801	138.457	27.10	18.08	73.42	27.40417	9.075	17.3	200.64055	52.45664	4021	155.61595
10	Mh03	Maharashtra	Mahad	5	3.831	2892.031	128.209	32.62	18.08	73.42	27.40417	9.075	17.3	200.64055	52.45664	4021	155.61595
11	Mh04	Maharashtra	Mahad	5	3.350	1837.037	65.822	29.82	18.08	73.42	27.40417	9.075	17.3	200.64055	52.45664	4021	155.61595
12	Mh05	Maharashtra	Mahad	5	3.546	2207.407	45.664	31.61	18.08	73.42	27.40417	9.075	17.3	200.64055	52.45664	4021	155.61595
13	Mh06	Maharashtra	Mahad	5	3.803	2541.863	72.917	1.25	18.08	73.42	27.40417	9.075	17.3	200.64055	52.45664	4021	155.61595
14	Mh07	Maharashtra	Mahad	1	3.757	2624.916	35.867	32.18	18.08	73.42	27.40417	9.075	17.3	200.64055	52.45664	4021	155.61595
15	Mh11	Maharashtra	Katraj	1	4.071	3154.658	16.385	35.49	18.45	73.86	24.4	12.6	24.1	273.91937	52.28216	835	112.70583
16	Mh12	Maharashtra	Wadgoan Budrug	1	3.812	2817.957	36.543	35.78	18.74	73.64	24.39167	12.66667	24.1	263.64432	52.55878	1637	145.4496
17	Mh13	Maharashtra	Kharadi	1	3.116	45.791	78.322	37.93	18.56	73.95	25.07917	13.14167	24.9	286.88339	52.77777	632	98.30233
18	Mh14	Maharashtra	Shirur	1	3.643	5172.615	26.633	43.82	18.82	74.38	25.39167	13.55	25.7	309.44107	52.72374	484	93.98095
19	Wb01	West Bengal	Kolkata	1	3.391	5643.996	38.007	28.00	22.54	88.34	26.91667	9.8	22.3	385.61838	43.94619	1632	98.95976
20	Wb02	West Bengal	Kolkata	1	3.824	3109.764	35.529	2.30	22.54	88.34	26.91667	9.8	22.3	385.61838	43.94619	1632	98.95976
21	Wb03	West Bengal	Kolkata	1	3.672	5996.409	42.399	4.28	22.54	88.34	26.91667	9.8	22.3	385.61838	43.94619	1632	98.95976
22	Wb04	West Bengal	Kolkata	4	3.736	7929.068	57.376	34.93	22.54	88.34	26.91667	9.8	22.3	385.61838	43.94619	1632	98.95976
23	Wb31	West Bengal	Kamalgazi	1	3.281	1639.506	203.640	1.73	22.26	88.23	26.34167	8.85	21.4	388.48676	41.35514	1640	100.18294
24	Wb33	West Bengal	Aamtala	1	3.176	72.727	227.012	1.93	22.22	88.23	26.39583	8.908333	21.4	386.63144	41.62773	1636	100.27528
25	Wb34	West Bengal	Kalikapur	1	3.566	1302.806	163.410	1.26	22.24	88.28	26.36667	8.95	21.5	386.27197	41.62791	1675	100.59586
26	Wb36	West Bengal	Gangajoyara	1	1.990	16.611	263.410	0.76	22.27	88.24	26.35	8.85	21.4	388.47604	41.35514	1640	100.13217
27	Wb32	West Bengal	Nayabad	1	3.887	2133.333	162.644	1.47	22.29	88.24	26.33333	8.85	21.4	389.61716	41.35514	1643	100.16805
28	Wb38	West Bengal	Ashoknagar	1	3.635	2223.120	315.326	22.41	22.5	88.37	26.875	9.566667	21.9	381.19131	43.68341	1659	99.01768
29	Wb39	West Bengal	Duttapukur	1	1.662	3040.180	223.372	39.14	22.46	88.32	26.54583	9.224999	21.7	387.84048	42.51152	1645	99.36141
30	Wb40	West Bengal	Karnamadhabpur	1	3.605	4158.025	205.747	20.94	22.43	88.24	26.27083	9.175	22	395.66666	41.70455	1610	99.469
31	Wb41	West Bengal	Rarjahat	1	4.074	2366.779	123.563	11.05	22.33	88.27	26.3375	8.974999	21.5	390.07648	41.74419	1656	100.41531
32	Ap01	Andhra Pradesh	Vishakapatnam	1	3.656	2384.736	19.538	41.46	17.69	83.22	27.85	8.06667	17	269.11472	47.45098	1017	93.36484
33	Ap02	Andhra Pradesh	Vishakapatnam	1	3.604	3444.220	18.074	45.05	17.69	83.22	27.85	8.06667	17	269.11472	47.45098	1017	93.36484
34	Tn01	Tamil Nadu	Kanchipuram	1	3.906	2216.386	106.363	12.19	12.82	79.7	28.42083	9.80833	18.4	274.32944	53.30616	1089	82.42749
35	Tn03	Tamil Nadu	Kanchipuram	1	3.762	1938.047	107.827	13.80	12.82	79.7	28.42083	9.80833	18.4	274.32944	53.30616	1089	82.42749
36	Goa4	Goa	Siolim	1	3.911	2360.045	24.718	30.25	15.62	73.78	27.1625	8.54167	14.2	127.8871	60.15258	3098	146.12427
37	Goa5	Goa	Siolim	1	3.274	483.502	200.282	24.47	15.62	73.78	27.1625	8.54167	14.2	127.8871	60.15258	3098	146.12427
38	Goa6	Goa	Siolim	1	3.819	4382.492	41.047	28.39	15.62	73.78	27.1625	8.54167	14.2	127.8871	60.15258	3098	146.12427
39	Goa7	Goa	Siolim	1	3.174	12.121	41.385	26.73	15.62	73.78	27.1625	8.54167	14.2	127.8871	60.15258	3098	146.12427
40	Goa8	Goa	Siolim	1	3.782	2728.171	51.520	32.65	15.62	73.78	27.1625	8.54167	14.2	127.8871	60.15258	3098	146.12427
41	Goa9	Goa	Siolim	1	3.925	1163.636	54.673	36.27	15.62	73.78	27.1625	8.54167	14.2	127.8871	60.15258	3098	146.12427
42	Goa10	Goa	Siolim	1	3.815	3592.368	109.065	27.36	15.62	73.78	27.1625	8.54167	14.2	127.8871	60.15258	3098	146.12427
43	Goa11	Goa	Siolim	1	3.929	7713.580	148.818	0.24	15.62	73.78	27.1625	8.54167	14.2	127.8871	60.15258	3098	146.12427
44	Goa12	Goa	Siolim	1	4.503	4647.363	117.962	23.69	15.62	73.78	27.1625	8.54167	14.2	127.8871	60.15258	3098	146.12427
45	Goa13	Goa	Siolim	1	3.833	1489.113	104.336	23.35	15.62	73.78	27.1625	8.54167	14.2	127.8871	60.15258	3098	146.12427
46	Goa14	Goa	Siolim	1	4.179	5325.253	165.259	26.58	15.62	73.78	27.1625	8.54167	14.2	127.8871	60.15258	3098	146.12427
47	Goa15	Goa	Siolim	1	3.948	3233.221	133.953	31.13	15.62	73.78	27.1625	8.54167	14.2	127.8871	60.15258	3098	146.12427
48	GA01	Goa	Bardez	1	2.309	6603.756	66.667	32.82	15.582711	73.813654	27.35417	9.075	15	131.48553	60.5	3118	146.0031
49	GA02	Goa	Bardez	1	3.117	4594.366	223.946	30.99	15.582711	73.813654	27.35417	9.075	15	131.48553	60.5	3118	146.0031
50	GA03	Goa	Bardez	1	2.438	4293.897	38.314	34.82	15.582711	73.813654	27.35417	9.075	15	131.48553	60.5	3118	146.0031
51	GA04	Goa	Bardez	1	2.328	6509.859	98.084	39.14	15.582711	73.813654	27.35417	9.075	15	131.48553	60.5	3118	146.0031
52	GA05	Goa	Bardez	1	2.320	960.563	224.904	36.17	15.582711	73.813654	27.35417	9.075	15	131.48553	60.5	3118	146.0031
53	GA06	Goa	Bardez	1	2.511	8838.498	180.460	32.66	15.582711	73.813654	27.35417	9.075	15	131.48553	60.5	3118	146.0031
54	GA07	Goa	Ponda	1	2.238	0.000	54.215	40.03	15.403001	74.019411	27.0125	8.858334	14.9	132.54718	59.4519	3056	144.2372
55	GA08	Goa	Ponda	1	2.481	9627.230	44.444	37.25	15.403001	74.019411	27.0125	8.858334	14.9	132.54718	59.4519	3056	144.2372
56	GA09	Goa	Quepem	1	2.908	3523.944	48.851	36.11	15.230334	74.064887	27.15417	8.941667	14.9	130.70537	60.0112	2964	141.4449
57	GA10	Goa	Quepem	1	3.367	5702.347	84.100	34.97	15.230334	74.064887	27.15417	8.941667	14.9	130.70537	60.0112	2964	141.4449
58	GA11	Goa	Bicholim	1	2.638	7636.620	68.966	39.82	15.5889	73.9654	27.2125	9.091667	15.3	139.18796	59.42266	3232	145.4919
59	GA12	Goa	Bicholim	1	4.195	4772.770	105.939	34.87	15.5889	73.9654	27.2125	9.091667	15.3	139.18796	59.42266	3232	145.4919
60	GA13	Goa	Ponda	1	2.298	3711.737	62.261	36.25	15.403001	74.019411	27.0125	8.858334	14.9	132.54718	59.4519	3056	144.2372
61	GA14	Goa	Ponda	1	2.584	9448.826	134.100	29.81	15.403001	74.019411	27.0125	8.858334	14.9	132.54718	59.4519	3056	144.2372
62	GA15	Goa	Ponda	1	2.571	5570.892	50.766	42.56	15.403001	74.019411	27.0125	8.858334	14.9	132.54718	59.4519	3056	144.2372
63	GA19	Goa	Modkai	1	2.165	0.000	105.939	40.57	15.4167	73.9419	27.17083	8.391666	13.9	125.99529	60.3717	2995	144.7338
64	Ka01	Karnataka	Hunsur	1	4.841	5929.068	61.430	47.25	12.31	76.28	23.85833	10.46667	17.7	182.7919	59.13371	827	82.27261
65	Ka04	Karnataka	Hunsur	1	2.907	6959.371	38.682	39.30	12.31	76.28	23.85833	10.46667	17.7	182.7919	59.13371	827	82.27261
66	Ka05	Karnataka	Mysore	1	3.535	6270.258	41.385	46.62	12.31	76.65	24.54167	10.2	16.9	175.01732	60.35502	700	85.35764
67	Ka06	Karnataka	Mysore	1	3.171	5230.976	39.471	37.18	12.31	76.65	24.54167	10.2	16.9	175.01732	60.35502	700	85.35764
68	Ka09	Karnataka	Mysore	1	3.363	1949.270	34.065	37.30	12.31	76.65	24.54167	10.2	16.9	175.01732	60.35502	700	85.35764
69	Ka10	Karnataka	Hunsur	1	3.849	2687.767	33.840	37.94	12.31	76.28	23.85833	10.46667	17.7	182.7919	59.13371	827	82.27261
70	Ka14	Karnataka	Hunsur	1	3.239	2308.418	42.736	40.03	12.31	76.28	23.85833	10.46667	17.7	182.7919	59.13371	827	82.27261
71	Ka17	Karnataka	Hunsur	1	3.334	1906.622	58.390	38.64	12.31	76.28	23.85833	10.46667	17.7	182.7919	59.13371	827	82.27261
72	Ka18	Karnataka	Hunsur	1	3.537	970.595	33.953	31.61	12.31	76.28	23.85833	10.46667	17.7	182.7919	59.13371	827	82.27261
73	Ka19	Karnataka	Hunsur	1	3.725	1269.136	32.827	34.08	12.31	76.28	23.85833	10.46667	17.7	182.7919	59.13371	827	82.27261
74	Ka20	Karnataka	Hunsur	1	3.702	741.639	20.327	41.48	12.31	76.28	23.85833	10.46667	17.7	182.7919	59.13371	827	82.27261
75	Ka26	Karnataka	Hunsur	1	3.468	1837.037	21.115	42.27	12.31	76.28	23.85833	10.46667	17.7	182.7919	59.13371	827	82.27261
76	Ka30	Karnataka	Hunsur	1	3.902	723.681	23.705	32.96	12.31	76.28	23.85833	10.46667	17.7	182.7919	59.13371	827	82.27261
77	Ka31	Karnataka	Dharwad	1	3.682	4532.884	50.394	23.14	15.38	75.11	24.7125	10.90833	19	193.14532	57.41227	851	86.49883
78	Ka32	Karnataka	Hunsur	1	3.304	1388.103	23.255	44.81	12.31	76.28	23.85833	10.46667	17.7	182.7919	59.13371	827	82.27261
79	Ka40	Karnataka	Belgaum	1	4.174	131.089	31.813	49.06	15.87	74.51	23.43333	10.95	19.8	191.30333	55.30304	2143	145.27356
80	KA02	Karnataka	Hunsur	1	3.349	12960.563	67.241	31.44	12.31	76.28	23.85833	10.466666	17.7	182.7919	59.13371	827	82.27261
81	KA03	Karnataka	Hunsur	1	3.339	8284.507	67.241	38.47	12.31	76.28	23.85833	10.466666	17.7	182.7919	59.13371	827	82.27261
82	KA05	Karnataka	Hunsur	1	3.684	7251.643	69.157	40.08	12.31	76.65	24.54167	10.2	16.9	175.01732	60.35502	700	85.35764
83	KA07	Karnataka	Mysore	1	2.857	13876.056	79.119	36.21	12.31	76.65	24.54167	10.2	16.9	175.01732	60.35502	700	85.35764
84	KA08	Karnataka	Muddnalli	1	3.372	8453.521	34.483	50.12	13.407	77.6962	23.62917	10.691667	19.9	241.14743	53.72697	717	88.5199
85	KA29	Karnataka	Hunsur	1	3.161	16472.300	85.632	37.42	12.31	76.28	23.85833	10.466666	17.7	182.7919	59.13371	827	82.27261
86	KAP1	Karnataka	Hunsur	1	4.232	8369.014	32.375	37.84	12.31	76.28	23.85833	10.466666	17.7	182.7919	59.13371	827	82.27261
87	KL02	Kerala	Mananthawady	1	3.336	5736.027	16.160	40.89	11.81	76.01	22.9	8.533334	14.4	146.72174	59.25926	2847	127.27962
88	KL03	Kerala	Kollam	1	3.478	11317.371	90.996	20.99	8.8932	76.6141	27.6	7.266666	10.6	92.09676	68.55347	2513	75.53255
89	KL04	Kerala	Kollam	1	3.424853425	11261.033	95.977	25.79	8.8932	76.6141	27.6	7.266666	10.6	92.09676	68.55347	2513	75.53255

Table B: Statistics of GWR and MLR (Global) Models: The table provies information on the results of the p-value and adjusted R2 value for Global MLR models and GWR models obtained for the enzymatic activities. The dependent variables (DV) such as PLA2 and protease were transformed into square root (Sq_activity), inverse (I_activity) and Logarthimic (L_activity) equations to improve model performance. The values from the untransformed models is also provided. The following independent variables (IV) were used in the models: Annual mean temperature: AMT, Temperature annual range: TAR, Annual mean diurnal temperature range: AMDTR, Isothermality: I, Temperature seasonality: TS, Annual precipitation: APNN and Precipitation seasonality: PS.

Predictor	Model_Type	Predictors_Used	Adjusted_R_Squared	AIC	Model_P_Value
PLA2	Global	AMT, AMDTR, TAR, TS, I, APNN, PS	0.21445349	983.118603	0.00032687
PLA2	GWR	AMT, AMDTR, TAR, TS, I, APNN, PS	0.21953617	727.969822	NA
PLA2	Global	AMT, AMDTR, TAR, I, APNN, PS	0.22376447	981.149435	0.00013361
PLA2	GWR	AMT, AMDTR, TAR, I, APNN, PS	0.22798595	726.093037	NA
PLA2	Global	AMT, AMDTR, TAR, APNN, PS	0.23199048	979.280042	5.14E-05
PLA2	GWR	AMT, AMDTR, TAR, APNN, PS	0.23575238	724.271968	NA
PLA2	Global	AMDTR, TAR, APNN, PS	0.2346293	978.039599	2.31E-05
PLA2	GWR	AMDTR, TAR, APNN, PS	0.23663604	723.234883	NA
PLA2	Global	AMDTR, TAR, APNN	0.23433358	977.127247	1.06E-05
PLA2	GWR	AMDTR, TAR, APNN	0.23638311	722.317634	NA
PLA2	Global	AMDTR, TAR	0.2346588	976.130384	3.77E-06
PLA2	GWR	AMDTR, TAR	0.23597859	721.405717	NA
Sq_PLA2	Global	AMT, AMDTR, TAR, TS, I, APNN, PS	0.18095503	1981.23959	0.0013576
Sq_PLA2	GWR	AMT, AMDTR, TAR, TS, I, APNN, PS	0.18510027	1726.21695	NA
Sq_PLA2	Global	AMT, AMDTR, TAR, I, APNN, PS	0.19000908	1979.3423	0.00061443
Sq_PLA2	GWR	AMT, AMDTR, TAR, I, APNN, PS	0.19370485	1724.36423	NA
Sq_PLA2	Global	AMDTR, TAR, I, APNN, PS	0.19861685	1977.47024	0.00025458
Sq_PLA2	GWR	AMDTR, TAR, I, APNN, PS	0.20112709	1722.61996	NA
Sq_PLA2	Global	AMDTR, I, APNN, PS	0.20518881	1975.80324	0.00010215
Sq_PLA2	GWR	AMDTR, I, APNN, PS	0.20737297	1720.98727	NA
Sq_PLA2	Global	AMDTR, I, APNN	0.21073416	1974.23339	3.68E-05
Sq_PLA2	GWR	AMDTR, I, APNN	0.21273264	1719.43669	NA
Sq_PLA2	Global	AMDTR, I	0.21926792	1972.3068	8.88E-06
Sq_PLA2	GWR	AMDTR, I	0.22094562	1717.54429	NA
I_PLA2	Global	AMT, AMDTR, TAR, TS, I, APNN, PS	0.2005038	-500.74813	0.00059857
I_PLA2	GWR	AMT, AMDTR, TAR, TS, I, APNN, PS	0.20748083	-756.09928	NA
I_PLA2	Global	AMT, TAR, TS, I, APNN, PS	0.21013132	-502.73433	0.00025038
I_PLA2	GWR	AMT, TAR, TS, I, APNN, PS	0.21554308	-757.91727	NA
I_PLA2	Global	AMT, TS, I, APNN, PS	0.21037663	-503.68318	0.00014645
I_PLA2	GWR	AMT, TS, I, APNN, PS	0.21524935	-758.80515	NA
I_PLA2	Global	TS, I, APNN, PS	0.19714491	-503.13828	0.00015141
I_PLA2	GWR	TS, I, APNN, PS	0.20021861	-758.05072	NA
I_PLA2	Global	TS, APNN, PS	0.18039081	-502.24686	0.0001721
I_PLA2	GWR	TS, APNN, PS	0.18188281	-756.98008	NA
I_PLA2	Global	TS, APNN	0.12730924	-497.62084	0.0010658
I_PLA2	GWR	TS, APNN	0.13071451	-752.53986	NA
L_PLA2	Global	AMT, AMDTR, TAR, TS, I, APNN, PS	0.22292659	189.989966	0.00022447
L_PLA2	GWR	AMT, AMDTR, TAR, TS, I, APNN, PS	0.22893865	-65.272346	NA
L_PLA2	Global	AMT, AMDTR, TAR, I, APNN, PS	0.23212953	188.021678	9.02E-05
L_PLA2	GWR	AMT, AMDTR, TAR, I, APNN, PS	0.23713532	-67.131475	NA
L_PLA2	Global	AMT, AMDTR, TAR, APNN, PS	0.22174488	188.296046	8.49E-05
L_PLA2	GWR	AMT, AMDTR, TAR, APNN, PS	0.22576392	-66.735814	NA
L_PLA2	Global	AMT, TAR, APNN, PS	0.21933222	187.637408	5.05E-05
L_PLA2	GWR	AMT, TAR, APNN, PS	0.22291848	-67.343444	NA
L_PLA2	Global	TAR, APNN, PS	0.18157417	190.89442	0.00016225
L_PLA2	GWR	TAR, APNN, PS	0.18486537	-64.035263	NA
L_PLA2	Global	TAR, APNN	0.11371257	197.024977	0.00207198
L_PLA2	GWR	TAR, APNN	0.11807648	-57.985383	NA
Protease	Global	AMT, AMDTR, TAR, TS, I, APNN, PS	0.48782885	666.480266	4.44E-11
Protease	GWR	AMT, AMDTR, TAR, TS, I, APNN, PS	0.49020251	411.495777	NA
Protease	Global	AMDTR, TAR, TS, I, APNN, PS	0.49256352	664.745734	1.21E-11
Protease	GWR	AMDTR, TAR, TS, I, APNN, PS	0.49379139	409.959056	NA
Protease	Global	AMDTR, TAR, TS, APNN, PS	0.47131856	667.474827	2.22E-11
Protease	GWR	AMDTR, TAR, TS, APNN, PS	0.47419811	412.417691	NA
Protease	Global	TAR, TS, APNN, PS	0.46039116	668.36151	1.69E-11
Protease	GWR	TAR, TS, APNN, PS	0.46228837	413.476986	NA
Protease	Global	TS, APNN, PS	0.44180996	670.427869	2.02E-11
Protease	GWR	TS, APNN, PS	0.4442797	415.462151	NA
Protease	Global	APNN, PS	0.16160664	705.672942	0.00019008
Protease	GWR	APNN, PS	0.17143717	450.052152	NA
Sq_Protease	Global	AMT, AMDTR, TAR, TS, I, APNN, PS	0.48782885	543.100068	4.44E-11
Sq_Protease	GWR	AMT, AMDTR, TAR, TS, I, APNN, PS	0.49020251	288.115579	NA
Sq_Protease	Global	AMDTR, TAR, TS, I, APNN, PS	0.49256352	541.365536	1.21E-11
Sq_Protease	GWR	AMDTR, TAR, TS, I, APNN, PS	0.49379139	286.578858	NA
Sq_Protease	Global	AMDTR, TAR, TS, APNN, PS	0.47131856	544.094629	2.22E-11
Sq_Protease	GWR	AMDTR, TAR, TS, APNN, PS	0.47419811	289.037493	NA
Sq_Protease	Global	TAR, TS, APNN, PS	0.46039116	544.981312	1.69E-11
Sq_Protease	GWR	TAR, TS, APNN, PS	0.46228837	290.096787	NA
Sq_Protease	Global	TS, APNN, PS	0.44180996	547.047671	2.02E-11
Sq_Protease	GWR	TS, APNN, PS	0.4442797	292.081953	NA
Sq_Protease	Global	APNN, PS	0.16160664	582.292744	0.00019008
Sq_Protease	GWR	APNN, PS	0.17143717	326.671954	NA
I_Protease	Global	AMT, AMDTR, TAR, TS, I, APNN, PS	-0.0114595	133.321363	0.54360498
I_Protease	GWR	AMT, AMDTR, TAR, TS, I, APNN, PS	-0.0106242	-121.32322	NA
I_Protease	Global	AMT, AMDTR, TAR, TS, I, PS	0.00047955	131.356619	0.42653703
I_Protease	GWR	AMT, AMDTR, TAR, TS, I, PS	0.00124071	-123.28224	NA
I_Protease	Global	AMT, TAR, TS, I, PS	0.01170454	129.430261	0.31245161
I_Protease	GWR	AMT, TAR, TS, I, PS	0.01238647	-125.20223	NA
I_Protease	Global	AMT, TAR, TS, PS	0.02086403	127.667447	0.21896739
I_Protease	GWR	AMT, TAR, TS, PS	0.02152933	-126.96411	NA
I_Protease	Global	TAR, TS, PS	0.02853683	126.020537	0.14222994
I_Protease	GWR	TAR, TS, PS	0.02898021	-128.59115	NA
I_Protease	Global	TAR, TS	0.03254002	124.693976	0.08972177
I_Protease	GWR	TAR, TS	0.03285829	-129.90637	NA
L_Protease	Global	AMT, AMDTR, TAR, TS, I, APNN, PS	0.32835755	242.118623	1.21E-06
L_Protease	GWR	AMT, AMDTR, TAR, TS, I, APNN, PS	0.33071184	-12.764954	NA
L_Protease	Global	AMDTR, TAR, TS, I, APNN, PS	0.33016308	240.971086	5.59E-07
L_Protease	GWR	AMDTR, TAR, TS, I, APNN, PS	0.33160907	-13.792306	NA
L_Protease	Global	AMDTR, TAR, TS, I, APNN	0.3290278	240.200603	2.82E-07
L_Protease	GWR	AMDTR, TAR, TS, I, APNN	0.3304292	-14.556538	NA
L_Protease	Global	AMDTR, TAR, TS, I	0.30617218	242.247639	4.63E-07
L_Protease	GWR	AMDTR, TAR, TS, I	0.30784476	-12.538228	NA
L_Protease	Global	TAR, TS, I	0.30652508	241.255626	1.75E-07
L_Protease	GWR	TAR, TS, I	0.30816721	-13.526433	NA
L_Protease	Global	TAR, TS	0.30213434	240.858301	7.13E-08
L_Protease	GWR	TAR, TS	0.30393485	-13.942677	NA

Table C: Statistics of SLR models. The table provies information on the results of the T-test including the parameter estimator, t-value, p-value and adjusted R2 value for individual SLR model obtained for the enzymatic activities. The information on the Homoscedasticity, Linearity and Normality of the models is also provided, wherein, the green boxes indicate a passed test and the red boxes indicate failed test. The dependent variables (DV) such as PLA2, protease and LAAO activities were transformed into square root (S_activity), inverse (I_activity) and Logarthimic (L_activity) equations to improve model performance. The values from the untransformed models is also provided. The following independent variables (IV) were used in the models: Annual mean temperature: AMT, Temperature annual range: TAR, Annual mean diurnal temperature range: AMDTR, Isothermality: I, Temperature seasonality: TS, Annual precipitation: APN and Precipitation seasonality: PS.

DV	IV	Parameter Estimator	t-value	p-value	Adjusted R2	Homoscedasticity	Linearity	Normality
PLA2	TAR	2.717	2.049	0.0471	0.117			
PLA2	AMDTR	4.865	0.94	0.353	0.00874			
PLA2	TS	0.109	2.417	0.0203	0.1357			
PLA2	I	-2.028	-2.459	0.01834	0.125			
PLA2	APN	0.011	1.775	0.0836	0.073			
PLA2	AMT	7.14	1.938	0.0597	0.06468			
PLA2	PS	0.56	2.756	0.00876	0.178			
S_PLA2	TAR	0.152	1.822	0.0759	0.088			
S_PLA2	AMDTR	0.268	0.825	0.414	0.0008495			
S_PLA2	TS	0.006	2.152	0.0375	0.105			
S_PLA2	I	-0.114	-2.147	0.0379	0.094			
S_PLA2	APN	0.001	2.075	0.0445	0.085			
S_PLA2	AMT	0.448	1.897	0.0651	0.064			
S_PLA2	PS	0.033	2.677	0.01073	0.153			
I_PLA2	TAR	-0.0003257	-0.498	0.6213	-0.01447			
I_PLA2	AMDTR	-0.0004076	-0.175	0.862	-0.02378			
I_PLA2	TS	-0.00001451	-0.611	0.545	-0.01			
I_PLA2	I	0.0002311	0.539	0.593	-0.01495			
I_PLA2	APN	-0.000005202	-3.137	0.00319	0.09673			
I_PLA2	AMT	-0.002	-1.446	0.1559	0.025			
I_PLA2	PS	-0.0001296	-1.592	0.119	0.03088			
L_PLA2	TAR	0.034	1.438	0.158	0.049			
L_PLA2	AMDTR	0.058	0.644	0.52334	-0.009			
L_PLA2	TS	0.001	1.693	0.0982	0.063			
L_PLA2	I	-0.025	-1.663	0.104	0.053			
L_PLA2	APN	0.0002029	2.456	0.0185	0.09488			
L_PLA2	AMT	0.118	1.796	0.08	0.05644			
L_PLA2	PS	0.008	2.456	0.0185	0.116			
L	TAR	61.14	1.089	0.281	0.006014			
L	AMDTR	74.79	0.393	0.696	-0.01623			
L	TS	2.799	2.558	0.2789	0.01531			
L	I	-64.04	-1.45	0.153	0.02955			
L	APN	-0.0141	-0.062	0.9505	-0.01917			
L	AMT	167.2	0.949	0.3471	-0.003642			
L	PS	2.492	0.26	0.7962	-0.01793			
L_L	TAR	0.0243	0.829	0.4109	-0.006668			
L_L	AMDTR	-0.005835	-0.042	0.9666	-0.01917			
L_L	TS	0.001446	1.328	0.1898	0.009811			
L_L	I	-0.03282	-1.707	0.09375	0.02114			
L_L	APN	-1.50E-05	-0.112	0.9115	-0.019			
L_L	AMT	0.08727	0.907	0.3685	-0.005859			
L_L	PS	-0.0007423	-0.15	0.8813	-0.01887			
L_S	TAR	-0.000221	-0.753	0.4548	-0.00794			
L_S	AMDTR	-0.0005603	-0.458	0.6492	-0.01347			
L_S	TS	-1.01E-05	-0.991	0.3262	-0.003949			
L_S	I	0.0002003	1.031	0.3075	-0.002902			
L_S	APN	1.06E-06	0.721	0.4743	-0.00681			
L_S	AMT	0.0005612	0.634	0.5291	-0.01322			
L_S	PS	3.92E-05	0.83	0.4103	-0.008247			
L_I	TAR	-0.000221	0.455	0.4548	-0.00794			
L_I	AMDTR	-0.0005603	-0.458	0.6492	-0.01347			
L_I	TS	-1.01E-05	-0.991	0.3262	-0.003949			
L_I	I	0.0002003	1.031	0.307	-0.002902			
L_I	APN	1.06E-06	0.721	0.4743	-0.00681			
L_I	AMT	0.0005612	0.634	0.5291	-0.01322			
L_I	PS	3.92E-05	0.83	0.4103	-0.008247			
PA	TAR	0.6795	1.483	0.1442	0.06142			
PA	AMDTR	3.472	3.016	0.00399	0.1475			
PA	TS	0.0125	0.511	0.6119	-0.001762			
PA	I	-0.1586	-0.462	0.6458	-0.01185			
PA	APN	-0.004449	-3.459	0.001103	0.1471			
PA	AMT	-3.796	-3.152	0.002712	0.18			
PA	PS	-0.04237	-0.753	0.455	-0.009989			
PA_L	TAR	0.01184	0.326	0.7455	-0.01559			
PA_L	AMDTR	0.13843	1.408	0.1652	0.02378			
PA_L	TS	-0.0004759	-0.266	0.7912	-0.01539			
PA_L	I	0.01086	0.427	0.6709	-0.01366			
PA_L	APN	-0.0002182	-1.657	0.1036	0.04592			
PA_L	AMT	-0.17528	-1.772	0.08243	0.04987			
PA_L	PS	-0.003963	-0.931	0.3562	-0.005865			
PA_S	TAR	0.04639	0.826	0.4126	0.007136			
PA_S	AMDTR	0.3133	2.216	0.03119	0.07675			
PA_S	TS	0.0001528	0.053	0.9582	-0.01942			
PA_S	I	0.003582	0.088	0.9305	-0.01933			
PA_S	APN	-0.0004308	-2.451	0.01769	0.09111			
PA_S	AMT	-0.3752	-2.507	0.01542	0.1184			
PA_S	PS	-0.005157	-0.792	0.4319	-0.009518			
PA_I	TAR	-0.009391	-0.592	0.5563	-0.01149			
PA_I	AMDTR	-0.05307	-0.931	0.3564	0.0008706			
PA_I	TS	-1.91E-04	-0.299	0.7663	-0.01744			
PA_I	I	0.003916	0.365	0.7164	-0.01713			
PA_I	APN	8.42E-05	1.122	0.2669	0.01172			
PA_I	AMT	0.04862	1.032	0.3069	-0.002436			
PA_I	PS	2.74E-03	1.114	0.2704	0.001516			

#CODE FOR SLR

```
model_name = lm(dv~iv, data = "loaded data table")
#set of dv and iv given in Table S1
```

#CODE FOR MLR

```
model_name = lm(dv~{set of iv to be considered}, data = "loaded data table")
#set of dv and iv given in Table S1
```

#CODE FOR MODEL CHECK AND VALIDATION

```
library(psych)
library(lm.beta)
library(lmtest)
library(sandwich)
library(car)
library(tidyverse)
library(DHARMa)
#Read in dataset here

df <- read.delim("file_path")
name=names(df)
name

df %>%
  ggplot(aes(dv, iv))+
  geom_point()

#documentation:
#summaryR.lm = summary function for model. fails in high multicollinearity situation
#All_PLOT = Plots MLR model of indicated model
#PLOT_FUNCTION = For graphical equation visualisation
#All_PLOT_MLR = runs through models to yeild the best MLR model; runs step-wise
regression

summaryR.lm <- function(model, type=c("hc3", "hc0", "hc1", "hc2", "hc4"), ...){

  if (!require(car)) stop("Required car package is missing.")

  type <- match.arg(type)
  V <- hccm(model, type=type)
  sumry <- summary(model)
  table <- coef(sumry)
  table[,2] <- sqrt(diag(V))
  table[,3] <- table[,1]/table[,2]
  table[,4] <- 2*pt(abs(table[,3]), df.residual(model), lower.tail=FALSE)
```

```
  sumry$coefficients <- table
  p <- nrow(table)
  hyp <- cbind(0, diag(p - 1))
  sumry$fstatistic[1] <- linearHypothesis(model, hyp,white.adjust=type)[2,"F"]

  print(sumry)
  cat("Note: Heteroscedasticity-consistent standard errors using adjustment", type,
"\n")

}


All_PLOT_MLR <- function(data, dependent_var, base_path, folder_name) {

  # Load necessary libraries
  library(ggplot2)
  library(MASS)

  # Automatically extract independent variables
  independent_vars <- setdiff(names(data), dependent_var)

  # Construct the full path with the folder name
  base_path <- paste0(base_path, "/", folder_name)
  if (!dir.exists(base_path)) {
    dir.create(base_path, recursive = TRUE)
  }

  # Full model with all predictors
  full_model <- lm(as.formula(paste(dependent_var, "~", paste(independent_vars,
collapse = "+"))), data = data)

  # Stepwise regression using AIC
  stepwise_model <- stepAIC(full_model, direction = "both")

  # Directory creation for results
  directory_path <- paste0(base_path, "/Stepwise_Regression")
  if (!dir.exists(directory_path)) {
    dir.create(directory_path, recursive = TRUE)
  }

  # Generate a plot with the final model predictors
  predictors <- all.vars(formula(stepwise_model))
  predictors <- setdiff(predictors, dependent_var) # To get only independent vars

  # Plot for each significant independent variable
  for (var in predictors) {
    pdf(file = paste0(directory_path, "/SLR_", var, ".pdf"))
    print(
```

```
    ggplot(data, aes_string(x = dependent_var, y = var)) +
      geom_point() +
      geom_smooth(method = "lm", se = TRUE) +
      labs(
        x = dependent_var,
        y = var,
        title = paste("Scatter plot of", dependent_var, "vs", var)
      )
  )
  dev.off()
}

# Saving regression analysis
sink(file = paste0(directory_path, "/Stepwise_Regression_Result.txt"))
print(summary(stepwise_model))
sink(NULL)
PLOT_FUNCTION(stepwise_model)
regression_analysis(formula = stepwise_model, data = data, path = base_path)
return(stepwise_model)
}


PLOT_FUNCTION = function(model) {
  coefficients <- coef(model)

  # Ordering coefficients by their absolute values (excluding intercept)
  ordered_vars <- order(abs(coefficients[-1]), decreasing = TRUE)
  ordered_coefficients <- coefficients[ordered_vars + 1]  # +1 to account for
intercept

  # Building the equation
  equation <- paste("Activity =", round(coefficients[1], 2),
                    paste(sprintf("%+.2f*%s", ordered_coefficients,
                                  names(ordered_coefficients)), collapse=" "))

  print(equation)
}


All_PLOT <- function(data, dependent_var, base_path, folder_name) {

  # Load necessary libraries
  library(ggplot2)

  # Automatically extract independent variables
  independent_vars <- setdiff(names(data), dependent_var)

  results <- list() # To store regression results for each independent variable
```

```
  # Construct the full path with the folder name
  base_path <- paste0(base_path, "/", folder_name)
  if (!dir.exists(base_path)) {
    dir.create(base_path, recursive = TRUE)
  }

  for (var in independent_vars) {

    # Define the regression formula
    formula_str <- as.formula(paste(dependent_var, "~", var))

    # Directory creation for each independent variable
    directory_path <- paste0(base_path, "/", var)
    if (!dir.exists(directory_path)) {
      dir.create(directory_path, recursive = TRUE)
    }

    # Scatter plot with regression line
    pdf(file = paste0(directory_path, "/SLR.pdf"))
    print(
      ggplot(data, aes_string(x = dependent_var, y = var)) +
        geom_point() +
        geom_smooth(method = "lm", se = TRUE) +
        labs(
          x = dependent_var,
          y = var,
          title = paste("Scatter plot of", dependent_var, "vs", var)
        )
    )
    dev.off()

    # Perform regression analysis and save output
    regression_analysis(formula = formula_str, data = data, path = directory_path)
  }

  return(results) # Returns a list of regression results for each independent variable
}


regression_analysis <- function(formula, data, path, confidence = 0.95, digits = 3) {

  # Load necessary libraries
  library(olsrr)
  library(jtools)
  library(moments)

  # Regression
```

```
reg.fit <- lm(formula, data = data)

# Data for linearity check

# Extract the independent variables from the regression model
independent_vars <- all.vars(formula(reg.fit))

# Subset the data for pairs plot
daten.plot <- data[independent_vars]

# Now plot with pairs function
pairs(daten.plot, pch = 19, lower.panel = NULL)

# 1 Regression output
# 1.1 Unstandardized results
#sink(file=paste0(path, "/SLR_Results.txt"))
#print(summaryR.lm(reg.fit))
#sink(NULL)  # Close the sink

# 1.2 Standardized results
sink(file=paste0(path, "/SLR_S_Results.txt"))
print(summ(reg.fit, scale=TRUE, transform.response = TRUE, digits=digits))
sink(NULL)

# 2 Regression diagnostics

# 2.1 Homoskedasticity
pdf(paste0(path, "/Homoskedasticity.pdf"))
ols_plot_resid_fit(reg.fit)
dev.off()

sink(file=paste0(path, "/Homoskedasticity.txt"))
print(ols_test_breusch_pagan(reg.fit))
sink(NULL)

# 2.2 Normality of the residuals
pdf(paste0(path, "/Norm_resid.pdf"))
ols_plot_resid_hist(reg.fit)
dev.off()

pdf(paste0(path, "/QQ_Plot.pdf"))
ols_plot_resid_qq(reg.fit)
dev.off()

sink(file=paste0(path, "/Shapiro.txt"))
print(shapiro.test(reg.fit$residuals))
sink(NULL)
```

```
  sink(file=paste0(path, "/SK.txt"))
  print(agostino.test(reg.fit$residuals))
  print(anscombe.test(reg.fit$residuals))
  sink(NULL)

  # 2.3 Linearity
  pdf(paste0(path, "/Scatter_plot.pdf"))
  pairs(daten.plot, pch = 19, lower.panel = NULL)
  dev.off()

  sink(file=paste0(path, "/Raintest.txt"))
  print(raintest(reg.fit))
  sink(NULL)

  # 2.5 Outlier diagnostics
  pdf(paste0(path, "/Residuals.pdf"))
  ols_plot_resid_stud(reg.fit)
  dev.off()

  pdf(paste0(path, "/CookDistance.pdf"))
  ols_plot_cooksd_chart(reg.fit)
  dev.off()

  pdf(paste0(path, "/Outliers.pdf"))
  ols_plot_resid_lev(reg.fit)
  dev.off()

  pdf(paste0(path, "/influence.pdf"))
  ols_plot_dfbetas(reg.fit)
  dev.off()

  return(reg.fit)
}
robust_scale <- function(x) {
  median_x <- median(x, na.rm = TRUE)
  iqr_x <- IQR(x, na.rm = TRUE)
  (x - median_x) / iqr_x
}
```

#CODE FOR PREDICTION MAP

```
install.packages("terra")
install.packages("rasterVis")
library(terra)
library(rasterVis)

# Define the geographic extent of India
# These are approximate boundaries and can be refined
```

```
xmin <- 68.1  # Minimum longitude
xmax <- 97.4  # Maximum longitude
ymin <- 6.75  # Minimum latitude
ymax <- 35.7  # Maximum latitude
india_extent <- ext(xmin, xmax, ymin, ymax)

# Load and clip bioclimatic layers
bioclim_layers <- list()
#in "for(i in 1:7)", in place of 7 insert number of abiotic factors to use
for (i in 1:7) {
  layer <- rast(paste0("insert file path", i, ".tif"))
  clipped_layer <- crop(layer, india_extent)
  bioclim_layers[[i]] <- clipped_layer
}
bioclim_stack <- rast(bioclim_layers)

intercept = #value
AMT = #value
MDR = #value
TAR =  #value
TS = #value
I = #value
AP = #value
PS = #value
#input other parameters if needed
coefs <- c(AMT, MDR, I, TS, TAR, AP, PS) #input other parameters if needed

bioclim_values <- as.data.frame(values(bioclim_stack))
predicted_values_"<insert toxin/enzyme name" <- (intercept +
rowSums(sweep(bioclim_values, 2, coefs, `*`))) #remember to change the equation
if data is transformed
pred_raster <- rast(nrows=nrow(bioclim_layers[[1]]),
ncols=ncol(bioclim_layers[[1]]), ext=india_extent)
values(pred_raster) <- predicted_values_"<insert toxin/enzyme name"

levelplot(pred_raster, col.regions=colorRampPalette(c("blue", "green", "yellow",
"red")),
          xlab="Longitude", ylab="Latitude", main="<"insert main graph title)
```

#Code for evaluation Global and GWR model performances (Replace enzymatic values with target of interest, here given with example of PLA2.)

```
# Required libraries
library(sp)
library(spgwr)
```

```
# Create the missing variables (ensure these variables exist)
df_PLA2$Sq_PLA2 <- df_PLA2$PLA2^2        # Square of PLA2
df_PLA2$I_PLA2 <- 1 / df_PLA2$PLA2       # Inverse of PLA2
df_PLA2$L_PLA2 <- log(df_PLA2$PLA2)      # Log of PLA2

# List of predictor variables
predictor_list <- c("PLA2", "Sq_PLA2", "I_PLA2", "L_PLA2")
predictors <- c("AMT", "MDR", "TAR", "TS", "I", "APNN", "PS")

coordinates(df_PLA2) <- ~Long + Lat

# Initialize an empty dataframe to store Adjusted R-squared, AIC, and model p-
values
adjusted_rsq_df <- data.frame(
  Predictor = character(),
  Model_Type = character(),
  Predictors_Used = character(),
  Adjusted_R_Squared = numeric(),
  AIC = numeric(),
  Model_P_Value = numeric(),
  RMSE = numeric(),
  stringsAsFactors = FALSE
)

# Function to fit the global model and calculate Adjusted R-squared, AIC, and F-test
p-value
fit_global_model <- function(response_var, predictors) {
  formula <- as.formula(paste(response_var, "~", paste(predictors, collapse = "+")))
  model <- lm(formula, data = df_PLA2@data)  # Use only the data part
  adj_r_squared <- summary(model)$adj.r.squared
  aic <- AIC(model)

  # Extract F-test p-value for the model
  f_stat <- summary(model)$fstatistic
  model_p_value <- pf(f_stat[1], f_stat[2], f_stat[3], lower.tail = FALSE)  # F-test p-
value

  # Calculate RMSE for Global model
  residuals <- df_PLA2@data[[response_var]] - predict(model)
  rmse <- sqrt(mean(residuals^2))

  return(list(model = model, adj_r_squared = adj_r_squared, aic = aic,
model_p_value = model_p_value, rmse = rmse))
}

# Function to fit the GWR model and calculate Adjusted R-squared and AIC
fit_gwr_model <- function(response_var, predictors) {
  formula <- as.formula(paste(response_var, "~", paste(predictors, collapse = "+")))
```

```
  gwr_model <- gwr(formula, data = df_PLA2, bandwidth = 50)

  # Extract local coefficients
  local_intercept <- gwr_model$SDF$`(Intercept)`
  local_coefficients <- as.data.frame(gwr_model$SDF[, predictors])

  # Calculate predictions
  gwr_predictions <- local_intercept
  for (var in predictors) {
    gwr_predictions <- gwr_predictions + local_coefficients[[var]] *
df_PLA2@data[[var]]
  }

  # Calculate Adjusted R-squared for GWR
  residuals <- df_PLA2@data[[response_var]] - gwr_predictions
  ss_res <- sum(residuals^2)
  ss_tot <- sum((df_PLA2@data[[response_var]] -
mean(df_PLA2@data[[response_var]]))^2)
  n <- nrow(df_PLA2@data)
  p <- length(predictors)
  adj_r_squared <- 1 - (1 - (1 - ss_res / ss_tot)) * (n - 1) / (n - p - 1)

  # Calculate AIC for GWR
  aic <- n * log(ss_res / n) + 2 * (p + 1)

  # Calculate RMSE for GWR model
  rmse <- sqrt(mean(residuals^2))

  # Placeholder for model p-value for GWR
  model_p_value <- NA  # GWR does not provide a global p-value

  return(list(adj_r_squared = adj_r_squared, aic = aic, model_p_value =
model_p_value, rmse=rmse))
}

# Loop over each predictor variable in predictor_list
for (response_var in predictor_list) {
  print(paste("Processing predictor:", response_var))  # Debugging output to ensure
loop is running

  current_predictors <- predictors  # Reinitialize the predictors for each iteration
  best_global_adj_rsq <- -Inf
  best_gwr_adj_rsq <- -Inf
  best_global_model <- NULL
  best_gwr_predictors <- NULL

  while (length(current_predictors) > 1) {  # Continue until only one predictor
remains
```

```
# Fit Global model
global_results <- fit_global_model(response_var, current_predictors)
global_model <- global_results$model
global_adj_rsq <- global_results$adj_r_squared
global_aic <- global_results$aic
global_p_value <- global_results$model_p_value
global_rmse <- global_results$rmse

# Fit GWR model
gwr_results <- fit_gwr_model(response_var, current_predictors)
gwr_adj_rsq <- gwr_results$adj_r_squared
gwr_aic <- gwr_results$aic
gwr_p_value <- gwr_results$model_p_value  # Placeholder
gwr_rmse <- gwr_results$rmse

# Track Adjusted R-squared, AIC, and model p-values
adjusted_rsq_df <- rbind(adjusted_rsq_df, data.frame(
  Predictor = response_var,
  Model_Type = "Global",
  Predictors_Used = paste(current_predictors, collapse = ", "),
  Adjusted_R_Squared = global_adj_rsq,
  AIC = global_aic,
  Model_P_Value = global_p_value,
  RMSE = global_rmse,
  stringsAsFactors = FALSE
))

adjusted_rsq_df <- rbind(adjusted_rsq_df, data.frame(
  Predictor = response_var,
  Model_Type = "GWR",
  Predictors_Used = paste(current_predictors, collapse = ", "),
  Adjusted_R_Squared = gwr_adj_rsq,
  AIC = gwr_aic,
  Model_P_Value = gwr_p_value,
  RMSE = gwr_rmse,
  stringsAsFactors = FALSE
))

# Update best models if better Adjusted R-squared found
if (global_adj_rsq > best_global_adj_rsq) {
  best_global_adj_rsq <- global_adj_rsq
  best_global_model <- global_model
}

if (gwr_adj_rsq > best_gwr_adj_rsq) {
  best_gwr_adj_rsq <- gwr_adj_rsq
  best_gwr_predictors <- current_predictors
```

```
    }

    # Remove least significant predictor from the Global model
    p_values <- summary(global_model)$coefficients[-1, 4]  # Exclude intercept's p-
value
    least_significant_predictor <- names(p_values)[which.max(p_values)]

    current_predictors <- setdiff(current_predictors, least_significant_predictor)  #
Remove least significant predictor
  }
}

# Save the data to a CSV file
write.csv(adjusted_rsq_df, "PLA2_adjusted_R_AIC_and_FtestPvalues_new.csv",
row.names = FALSE)

# Optionally, you can also assign the data frame to a variable
PLA2_adjusted_R <- adjusted_rsq_df
```
