# Supplementary material for: Significant Serpents: Predictive Modelling of Bioclimatic Venom Variation in Russell’s Viper
Source: PLoS Negl Trop Dis. 2025 Apr 10;19(4):e0012949. doi: 10.1371/journal.pntd.0012949 (PMC11984747; doi:10.1371/journal.pntd.0012949)
Supplement: Attachment — Submitted filename: Significant Serpents_Reviewer response.docx [file pntd.0012949.s003.docx]

**Reviewer responses**

**Reviewer 1**

**The objectives are well articulated and the design is slightly adequate, but the population is neither clearly described neither sampled to a satisfactory extent so as to meet the study aims.**

We thank the reviewer for the kind comments.

**I don't believe the sample size is sufficient for the hypotheses tested.**

**Some of the statistical analyses were deficient, and this is acknowledged by We but this was not taken into account to fine tune the analyses, or to evaluate the results shown.**

We would like to point out that the study model, *Daboia russelii*, falls under Schedule 1 of the Indian Wildlife Act. This makes it nearly impossible to collect biological samples from these animals. It is also difficult to cover an area of 6000 km and collect ‘enough’ samples for statistical analyses. It has taken over 10 years to sample in some regions, and this was before the laws were made more stringent. Despite this, we have sampled different biogeographic regions and collected **the most snake venom samples** reported in any study from the Indian subcontinent. In any case, we politely disagree that 115 venom samples are insufficient, but we have noted this as a limitation in the discussion section.

**I do not think that the analyses and results support the conclusions, mainly for two problems: The decision to not model spatial clustering inflates parameter estimates and p-values and the decision to not use generalised linear models do not allowed We to evaluate significance with the appropriate distribution of errors.**

**The relevance of the data are discussed and is relevant to public health, but as mentioned I do not think that the results as shown are reliable.**

We respect the reviewer's views, however, we respectfully disagree with them. Only valid linear models have been chosen and ranked in response to AIC. To account for spatial autocorrelation, we have now included GWR models, which show no large deviation in model parameters.

**Reviewer 2**

**There are areas with low records of data for this species. It would be interesting to check in which eco-regions of India do you have records and in which other you do not have records.**

This data is neither readily available nor reliable. The absence of data in many regions (e.g., northeast India) may be associated with difficulty navigating the terrain and finding snakes. As far as the distribution is concerned, this snake has been recorded in most parts of India.

**Also, the experimental setup for measuring toxicological effects is hard, so you should put that trade-off between having more samples across the country and having more samples to get a representation of the total distribution of this snake in India. In addition, it would be interesting to add the populations that you are covering in venom sampling for this species.**

We have highlighted which populations are covered in the study in Figure 1. We are refraining from concluding w.r.t. the distribution of this species given the aforementioned limitations.

**How can you be sure that pulled venoms don’t generate a bias in terms of intraspecific variation or onthogenic variation? Please add the information about the age/size of each snake used in the pull and how do you can be sure that onthogenic variation does not affect your results.**

We have isolated venoms from individuals where it was enough. However, sometimes, you don’t get ‘enough’ venoms from one individual, so the venoms from multiple individuals are pooled. Note that we have also collected venoms individually from each location. As mentioned in line 119 of the paper, only adult venoms were analysed. Hence, ontogenetic shifts will not affect our analyses and interpretations. Hence, this shouldn’t affect our analyses, especially considering that we haven’t noted intraspecific venom variation in *D. russelii* at such short distances in our previous studies (Senji Laxme et al., 2024).

**Please improve the simple and multiple lineal regression analysis to correct geographically your results. How do you know that you don’t have spatial autocorrelation? If so, how do you ensure that your patterns are product of climate variables instead of spatial contiguity of locations? You can use geographically weighted regressions, or Bayesian inference in mixed effect lineal models by adding spatial autocorrelation as a random effect in the intercept, see ICAR and BYM models.**

We thank the reviewer for the recommendations. The suggested analysis regarding GWR has been performed, which is appended to the supplementary files (Table S2). Since no significant spatial autocorrelation was observed, no further models were used.

**The x-axis labels in the figure 2 are not clear. Is it a variable? Are you presenting histograms for each region? Why do you have a scale from 1 to >80 there? Please add the label of x-axis if it is a variable, or remove labels and thicks if it is only the number of the sample that you are plotting.**

We have now clarified the x-axis as follows in the figure legend. We have also added this information to the figure.

**Figure 2. Enzymatic activities of Russell’s viper venoms.** Bar graphs depicting the (A) PLA_2_, (B) protease and (C) LAAO activities of *D. russelii* venoms from across India are shown, where the x-axis represents sample IDs in Table S1, while the y-axis represents specific activities (nmol/mg/min).

**There is a mistake in table 1, in equation for PS. I can only see squares instead of the variables that are used in the expression.**

We thank the reviewer for the correction.

**Please change the name of the last column because significance can be confounded with a statistical analysis that shows that this variable modifies your toxicological variables significantly.**

The last column has been changed to “biological relevance”.

**It is not clear the difference between your SLR and MLR. Why are you saying that you used SLR but you talk about the effects of multiple climatic variables? I understand that you are explaining each variable independly in different models, but it is only clear after looking at S2 table. Please enhance the way as you describe this section and also in methods: You are conducting different SLR for each independent variables and each SLR is independent of others SLR.**

We thank the reviewer for the comment. SLR models (now in Table S3) were used to ascertain the independent contributions of each bioclimatic parameter, while MLR was used to understand their combined effect on venom variation. The paragraph has been formatted to best represent the data.

**In MLR it is not clear if you used also interactions between your variables. As a consequence, you are showing a lot of information of several models that are not good explaining your data, thus it is difficult to understand which model are you selecting as your best model to generate predictive maps. If you use GWR you can compare between all the possible combinations of the independent variables (Also evaluating each variable by itself) in different models and compare them by AIC or BIC, which punish the predictive capacity of the model by the number of parameters. As a consequence, you can select the model that explain best your data without doing overfitting by having a lot of parameters, and the use this model to generate your spatial predictions.**

We thank the reviewer for the suggestion. A GWR analysis was performed on the sample set, and no significant impact of spatial autocorrelation was observed. Even though GWR models provided lower AIC values in certain cases, the global model was used for spatial prediction. Since the GWR did not significantly improve model performance, the relation between the predictor and response variable would be spatially homogenous (Table S2).

We would also like to clarify that the models do not consider interactions, keeping each parameter separate. The model used to generate spatial predictions was mentioned in the selected model in Figure 3 in a blue highlight (a colour code is provided). As depicted in Figure 3, the model with a suitable AIC value was selected.

**The figure with the extrapoaltions done using your best model is confousing because the colorbar have above Longitude. Please include the label and the units for this colorbar, which I think should be the units of your dependent variables.**

We agree with the reviewer. The image has been relabeled accordingly.

**Discussion:**

**Your first paragraph is large and you are talking about different topics. I liked a lot the analysis of the neglection of environmental factors driving phenotypic characteristics of venom, which is crucial, but then you talk about the representativity of your data. Split this paragraph in two paragraphs.**

We thank the reviewer for the kind comment, and the paragraphs have now been shortened and edited accordingly.

**When you talk about the number of venom samples that you got, please refer to what I told you in methodology: You can have a lot of samples, but spatially they could be not so representative (i.e. if you are missing samples on different ecosystems/eco-regions/life zones). Please enhance the methodology by adding this so you can discuss of the real representation of your data over India.**

We have collected samples from various places in a given biogeography. So, we can’t miss samples from ecosystems/ecoregions. We have collected venoms only from adults, so ontogeny can’t affect our interpretations. Moreover, in addition to the existing linear models, we added GWR models to see if there was any faulty interpretation due to spatial autocorrelation (Table S2).

**Please include about why did you not use anthropogenic intervention such as human footprint in your analysis. Can snakes sharing areas with humans during several years have different phenotypes in their venom?**

We thank the reviewers for the idea, however, this is beyond the scope of the present study design.

**What about climate change? If climate is moduling venom phenotipic, what can we expect with the ongoing climate change and it effects in India? Can venom became more lethal? please add a paragraph discussing about climate change.**

We haven’t correlated environment with lethality and hence can’t comment on that in this paper. It is also ethically, financially, and strategically challenging to generate toxicity data for many samples. We do show the effect of the environment on toxin activities. Interpretations w.r.t. climate change should be made w.r.t. how these abiotic factors affect activities.

**I found your discussion well documented, and you touched really interesting points. I think that you just need more robustness in the methods to support strongly your discussion. Congrats it is a nice job!**

Thank you for the kind comment. We have incorporated the suggestions to increase the robustness of the study.

**The subject of this article is highly relevant, and will provide useful information for public health authorities, medical practitioners and ecologists alike. My expertise falls within the spatial analysis side of this submission and this is what I have evaluated. A comprehensive review I think will need to include the opinion of molecular biology experts to.**

Thank you for the kind comment.

**“Parameter selection and data extraction”**

**I believe that referring to climatic data used as independent variables or covariates as “Parameters” is inadequate.**

Thanks for the suggestion. We have now renamed the paragraph to “Selection of bioclimatic variables and data extraction”.

**Its mentioned that bioclimatic data represent climate from 1895-2009. While adequate, we should be given additional information regarding the following aspects:**

**1.Did you use the bioclimatic variables calculated with the average of temperature and precipitation of the entire period?**

**2. Or did you use the climatic conditions estimated for each year by matching the date the venom samples were collected?**

The data used represents the historical data over a period of time, and has been averaged across months to give the best representative and comprehensive dataset (provided in Table S1).

**3.If such a long term average climatologies were used, there should be a justification , as you later suggest that you avoided the variables that represent seasonality. Including these variables may be important even if you do not focus on spatio-temporal patterns because temporal variability is inherent to biological processes.**

The historical values are representative of that region's bioclimatic characteristics. In studies such as Zancolli et al, 2019 and Smith et al, 2023, such values have been used as prediction time points. Based on our previous observations in Senji Laxme et al., 2024, seasonality did not play a major role in determining the venom phenotypes of *D. russelii.* Hence, historic abiotic factor information was used to predict the correlation between environmental conditions and venom variations across large time scales.

**4.To extract the raster values using locations of venom samples, were these locations simple points or do they correspond to polygons?**

Simple points were used to extract the raster values since the climatic conditions were fairly uniform in a region.

**“Linear regression models”**

**How exactly was enzymatic activity measured and so what are the units analysed? This is critical for the reader to understand the meaning of the models. Apologies if this was explained in detail previously, but given the amount of technical terms I could not identify this information in a straightforward way, so it would be useful to describe succinctly in this section**.

We measured the enzymatic assay using a colourimetric assay, which has been explained in detail in the methods section of the paper (section 2.2-2.4). The method used in tabulating enzymatic values has been provided in the methods section of the paper.

**At this point we should be given an idea of where were the samples obtained from, to assess how complete is the spatial coverage of the samples. This should be as complete as possible, because the use you are looking for these analyses is spatial prediction.**

**I also believe that, given the spatial nature off your analyses you should have used a spatially explicit analytical method to avoid pseudo-replication due to spatial autocorrelation.**

The details for sampling locations are provided in Table S1: Sampling location and bioclimatic variables. We have now included additional analysis to account for spatial autocorrelation by invoking GWR models, which have been included in the paper (Table S2).

**Ok, so the units of the data are mg/nmol/min, meaning that the units are min-1, which makes the data more suitable for a gamma model.**

We thank the reviewer for this suggestion. However, We would like to point out that the enzymatic values are absolute values of enzymatic activities. The minute parameter is a residual effect unit of the calculations required to get the specific activity of the enzyme and not a function of the biogeographical distribution of the species. Hence, we feel that the reasoning for the usage of a gamma inverse model is not relevant to this analysis.

**By inspection of the spatial distribution of the sampling points, there appear to be very large gaps and some point clusters. This would invalidate the analyses and the statistical inferences regarding the effect of the bioclimatic variables cannot be trusted unless point clustering is explicitly modelled.**

Kindly refer to the challenges associated with venom sampling, which are now pointed out in the X section.

**I agree that the predictions are very questionable. Some of the problems may be fixed if a more adequate type of model is used such as gamma, given the units of the data and spatial clustering. Accounting for spatial clustering tends to reduce the size of estimated coefficients and increase p-values.**

We agree on this point, however, to account for this, we have now included GWR models.

**MLR models**

**A further test would be to analyse the spatial correlation of model residuals.**

We agree and have now included GWR models to account for spatial correlation.

**Predictive mapping**

**I don’t think neither the spatial coverage of the d tata nor the methods make for models suitable for the intended use. One work around would be to show that the resulting maps can roughly predict enzymatic activity, using cross validation, for instance selecting data points as independent as possible. Alternatively, mapping may be restricted to data used and show interpolated values rather than using covariates with very weak effects on the response variable and potentially with extrapolation issues after projecting to the entire geographic extent of India.**

Venom as a phenotype is highly variable, and the causal reasons are yet to be understood. To date, our lab has shown the influence of environment and ontogeny on variation in the venom of the model system. We do not claim to explain the entire range of variation observed in the enzymatic data set, as there would be a definite impact of biotic factors, which also introduce these variations. The range of these biotic variations is unknown and would explain the variance in more detail. At present, considering the models observed, these are prediction models of the climatic component of enzymatic activity.
