# Supplementary material for: Significant Serpents: Predictive Modelling of Bioclimatic Venom Variation in Russell’s Viper
Source: PLoS Negl Trop Dis. 2025 Apr 10;19(4):e0012949. doi: 10.1371/journal.pntd.0012949 (PMC11984747; doi:10.1371/journal.pntd.0012949)
Supplement: Attachment — Submitted filename: Significant Serpentes_R2_Reviewer response.docx [file pntd.0012949.s004.docx]

We thank the reviewer and the editors for their kind suggestions. The changes and alterations have been noted, and applied wherever deemed necessary. Scientific rebuttal has been provided as well for points which required further clarification.

**METHODS**

Reviewer #2:

1. Please include explicitely which samples were pooled and which were not pooled. Include this as different colours in the dots presented in Figure 1.

It is not possible to show these in different colours as some samples in the same region are pooled, and others were collected individually. As many as 26 samples were collected in some regions, so highlighting them in different colours will make this figure confusing. This information is already provided in the Supplemental Table S1.

2. In PLA2, Protease and LAOO activities assay section, you are using the same quantity of mass of different venom and diluting it in the same quantity of NOB substrate. However, you said that you measured protein concentration per venom. I would recommend to clarify how can you do the analysis without including the possible differences in venom concentrations per each sample, which can cause different ammount of enzimatic activites instead of a variation in composition.

Here, the mass of the venom used in the biochemical analysis is based on the protein concentration of the venom and not the dry weight. Hence, the quantity of protein remains the same across all assays, which accounts for any possible enzymatic activity variation only due to composition. To dispel any confusion, we have explicitly mentioned that it is venom protein in the revised version of the manuscript.

3. It is not clear why did you removed seasonal data such as Max temperature at warmer month and the others. This data only uses the warmest month without assuming that that max temperature occured in the same month each year, of that the warmest mont was the same each year, which can be seasonal. Also, even if these variables talk about seasonality they are really important in the ecology of snakes: There are different ecological traits between rattlesnakes in U.S. (Seasonal temperature) and tropical rattlesnakes (Quite "constant" temperature). Although you don't have data for same snake per each time in the year (Which can be another great idea for another work to seek how "plastic" can venom expression be), a spatial area with high seasonality and another one without that seasonality can be really different and shape different condition. I recommend to include these variables in the models (In my experience, these variables shape really well difference of distribution between Bothrops vipers, because they define different ecological areas).

The temporal variation in sample collection restricts the usage of this particular parameter. The snake may not have been located at that point when the highest temperature in a particular month was recorded. To acknowledge the paradigm mentioned by the reviewer, we consider the Temperature Annual Range and the Mean Diurnal Range of Temperature separately to account for the seasonality in temperature conditions at a particular location, while averaging the variations over time. We accept that local models may reflect finer resolutions of variation, however, the model proposed in our paper reflects a wider spatial distribution, since local models are restricted by sampling size.

4. The statistical lineal models are well explained, and I find them really simple to understand which is really nice because you can explain the variation with basic models thus supporting really well your hypothesis. The selecion of GWR is also really nice for accounting for spatial autocorrelation. I found these sections nice.

We thank the reviewer for the kind comments.

# **Results**

1. Figure 1 and Figure 2: I liked both figures, but in figure 2 is kind of difficult to assign for each spatial label the spatial area in the map. Please include in Figure 2 each one of the areas that you refer in Figure 2 (PB, RJ, etc) as a vector so readers can locate easily in the map each area. Also, this can help to readers to determine which areas can be undersampled.

We thank the reviewer for the suggestion. It maybe pointed out, for the ease of a global audience, we have delimited the distribution into northern, western, eastern, central, southern and south-eastern Indian populations. These are representative of the political delimitations of the country, which do not coincide with the geographical features, and thus don't hold any bearing on the study.

However, following the reviewer’s suggestion, we have now added these locations in Figure 1.

2. As I told you in methodology, several variation between activities could be caused by different venom concentrations, which you measured but didn't use in the models. Thus, you cannot attribute your found differences to composition because they can be caused by concentrations. Maybe a way for looking at this without including concentration in your models is to perform correlation matrix analysis (Pearson or Spearman) between your enzimatic activities: You compute the correlation between all the samples for PLA2 activity with LAAO, then between PLA2 and Protease, and finally between LAAO and protease. With this analysis, if you find a strong correlation between activities it tells you that some samples have strong activities for all kind of enzimatic activites, whilst others have low activites but also for all, which can be telling you that venom concentration is the mechanism that is causing the differences. Another way for doing this is including in your lineal models (Indepently and also in the multiple lineal models) venom concentration.

We would like to inform the reviewer that the enzymatic data generated utilised protein concentrations of venom, and not the dry weight, which is exactly what has been suggested.

3. Please include explicitely that GWR results didn't show spatial autocorrelation, and that thanks to that you only used lineal models without any kind of spatial structure.

We thank the reviewer for the suggestion, and have included the line “ The analysis failed to identify any potential issues as a result of spatial autocorrelation.” in our manuscript in the materials and methods section.

4. The final step of performing these risk maps based on enzimatic activities is really clever! I think that including and correcting these possible bias, you can generate risk maps with more robustness and also understand deeper the mechanism behind this variation. This topic is fascinating, and reading and looking these results is really nice. I find very useful to include ecology to understand toxinological variation, it is a novel topic so I want to congrat you for this work.

We thank the reviewer for his kind comments.

# **Conclusions**

1. You attribute the changes in enzymatic activites with venom conposition, but given that you are not testing if differences are caused by different protein concentration in venoms you cannot conclude this. Please include concentration as told you before, so you can determine if differences are caused protein concentration (Which can be fascinating because it will imply a different way of variation by saving resources by producing venoms with not so much proteins, which are expensive. Thus, maybe for these populations evolution shapes that selecting forces makes for snakes more important to invest energy in other stuff rather than producing a high concentrated venom with higher enzymatic activities), or if they may be changed by venom composition (Which is another way that can be more related with different genetic expression).

Kindly refer to the answer above.

2. Please include a paragraph about the limitations of pooling venoms for these toxicological studies: You are addresing this issue as using most of the venoms at an individual level, but this is no acknowledged in the discussion. Please aslo explain explicitely that you avoided onthogenic shift by using only adults, but that if reproduction data is inexistent for ensuring that all individuals are adults based on body measurements such as SVL pooling must be done carefully. Also relate here to possible intersexual differences in venom composition and add the references.

We address this query in the limitations section of the paper, and add on this line for further measure: “In this study, we have also accounted for the influence of various ecological factors on venoms. For instance, Russell’s viper has not been documented to exhibit intersexual venom variation (Senji Laxme 2024). While ontogenetic shifts in venom profiles have been documented in this species (Senji Laxme 2024), we negated these effects by specifically sampling venoms from adult individuals.”

3. I would love to see a second part of the paper performing proteomic analyses between population to identify possible variations in protein composition, by including something that I found sometimes neglected in toxinology: The individual variation of venom composition. You had some pooled venoms which can have bias, but in general you are trying to go at an individual level. Several toxinologic studies pool venom, so you can get imformation about the averaged values of venom variables, but you don't get information about this dispersion: You cannot test if the found differences are significant.

We acknowledge the reviewer’s opinion and regard it as a possible future direction for this study.

# **Summary and General Comments**

This work account for a novel and fascinating topic: Looking at geographic variation of venon toxiconology by including statistics, thus having statistical tests to account for statistical significance in that variation. Several toxinologic studies uses pooled venoms and only refer to mean values to tell about differences, but this neglecet the intrinsic variation between venom in the comparet populations, so they cannot assure that the differences exist and are not caused by randomness.

We thank the reviewer for his kind comments.

The study have one limitation that can be addressed to generate a great work: 1. Measuring protein concentration of venom but not using it in the lineal models to account if the activities differences are caused by protein concentrations rather that venom composition

I suggest to correct this issues, or to account and aknowledge these important limitations in the paper, to generate a comprehensive study that address an important topic: ¿Why is venom variating between regions?

As mentioned before, we have considered protein concentration and not dry weight in our enzymatic assessment.

As a personal comment, I didn't like the title "Significant serpents". I think that this beginning is not related and is not highlighting the real importance of your work.

We respectfully disagree, and would like to follow through with our title, as we belief it does reflect the study in question.
